# Transparency, Documentation, and Open Science

**DOI:** 10.1093/geroni/igaa057.3162

**Published:** 2020-12-16

**Authors:** Derek Isaacowitz

**Affiliations:** Northeastern University, Boston, Massachusetts, United States

## Abstract

Some GSA journals are especially interested in promoting transparency and open science practices, reflecting how some subdisciplines in aging are moving toward open science practices faster than others. In this talk, I will consider the transparency and open science practices that seem most relevant to aging researchers, such as preregistration, open data, open materials and code, sample size justification and analytic tools for considering null effects. I will also discuss potential challenges to implementing these practices as well as reasons why it is important to do so despite these challenges. The focus will be on pragmatic suggestions for researchers planning and conducting studies now that they hope to publish later.

